# Eradication of bovine tuberculosis at a herd-level in Madrid, Spain: study of within-herd transmission dynamics over a 12 year period

**DOI:** 10.1186/1746-6148-8-100

**Published:** 2012-06-29

**Authors:** Julio Alvarez, Andres M Perez, Javier Bezos, Carmen Casal, Beatriz Romero, Sabrina Rodriguez-Campos, Jose L Saez-Llorente, Rosa Diaz, Jesus Carpintero, Lucia de Juan, Lucas Domínguez

**Affiliations:** 1 Instituto de Investigación en Recursos Cinegéticos IREC (CSIC-UCLM-JCCM), Ronda de Toledo s/n, 13071, Ciudad Real, Spain; 2 Center for Animal Disease Modeling and Surveillance (CADMS), Department of Medicine and Epidemiology, School of Veterinary Medicine, University of California, Davis, CA, 95616, USA; 3 CONICET/Facultad de Ciencias Veterinarias UNR, Boulevard Ovidio Lagos y Ruta 33, Casilda, Santa Fe, Argentina; 4 Centro VISAVET, Universidad Complutense de Madrid, Avda. Puerta de Hierro S/N, 28040, Madrid, Spain; 5 Subdirección General de Sanidad de la Producción Primaria, Dirección General de Recursos Agrícolas y Ganaderos, Ministerio de Medio Ambiente y Medio Rural y Marino, C/ Alfonso XII, 62, 28071, Madrid, Spain; 6 Área de Ganadería, Dirección General de Medio Ambiente, Consejería de Medio Ambiente, Vivienda y Ordenación del Territorio de la Comunidad de Madrid, C/ Ronda de Atocha 17, 28012, Madrid, Spain; 7 Departamento de Sanidad Animal, Facultad de Veterinaria, Universidad Complutense de Madrid, Avda. Puerta de Hierro S/N, 28040, Madrid, Spain

## Abstract

**Background:**

Eradication of bovine tuberculosis (bTB) through the application of test-and-cull programs is a declared goal of developed countries in which the disease is still endemic. Here, longitudinal data from more than 1,700 cattle herds tested during a 12 year-period in the eradication program in the region of Madrid, Spain, were analyzed to quantify the within-herd transmission coefficient (β) depending on the herd-type (beef/dairy/bullfighting). In addition, the probability to recover the officially bTB free (OTF) status in infected herds depending on the type of herd and the diagnostic strategy implemented was assessed using Cox proportional hazard models.

**Results:**

Overall, dairy herds showed higher β (median 4.7) than beef or bullfighting herds (2.3 and 2.2 respectively). Introduction of interferon-gamma (IFN-γ) as an ancillary test produced an apparent increase in the β coefficient regardless of production type, likely due to an increase in diagnostic sensitivity. Time to recover OTF status was also significantly lower in dairy herds, and length of bTB episodes was significantly reduced when the IFN-γ was implemented to manage the outbreak.

**Conclusions:**

Our results suggest that bTB spreads more rapidly in dairy herds compared to other herd types, a likely cause being management and demographic-related factors. However, outbreaks in dairy herds can be controlled more rapidly than in typically extensive herd types. Finally, IFN-γ proved its usefulness to rapidly eradicate bTB at a herd-level.

## Background

Bovine tuberculosis (bTB), caused by members of the *Mycobacterium tuberculosis* complex (mainly *M. bovis* and to a lesser extent *M. caprae*), is a zoonotic disease affecting cattle worldwide [1]. Test and slaughter programs, mostly based on skin testing using bovine PPD (single skin test) and in some countries also avian PPD (comparative skin test), have reduced bTB incidence and even achieved its eradication in certain countries and regions [2]. However and despite great efforts and resources invested in the fight against the disease, including eradication campaigns in place for >20 years, bTB is yet to be eradicated from many countries in the world [3,4]. Certainly, eradication programs face several challenges that impair their progress even in developed countries, including limitations on the sensitivity and specificity of the routine diagnostic tests, large herd sizes, animal movements, and the presence of wildlife reservoirs [5]. Herd type also seems to influence the risk for the disease, with dairy herds being at the highest risk, probably due to a combination of high within-herd animal density, which increases the frequency of animal contacts, and management practices that promote disease transmission [6,7], although contradictory results have been also reported [8]. Studies of bTB dynamics in infected herds are complicated by the disease’s long incubation period. For this reason, epidemiological models have been developed to quantify the within-herd transmission rate as a prerequisite to evaluate the impact of alternative control strategies in cattle [9,10] and deer [11]. However, epidemiological studies aimed at assessing the evolution of bTB control programs in a region and considering the impact of control strategies in different herd types have not been published in the peer reviewed literature.

In Spain, herd prevalence has decreased since the implementation of the National Eradication Program from 11.4% in 1986 to 1.65% in 2009 [12]. However, such improvement has not been homogeneous throughout the country and herd prevalence may be as low as <0.2% and as high as >5% depending on the region; for example, bTB herd prevalence in the autonomous community of Madrid (ACM) was 5.54% in 2009 [12], being the third largest prevalence recorded in the country. ACM, located in central Spain, currently holds a cattle population of approximately 1,500 herds and 100,000 animals including all productive types present in the country (around 85%, 8% and 7% of beef, dairy and bullfighting herds) of which all are exposed to a variety of epidemiological factors typically observed throughout Spain.

The study here was aimed at assessing the association between herd type and metrics of evolution of the bTB control program in ACM, including time to recover bTB free status and transmission rate, using data collected over >10 years. The influence that use of new diagnostic tests for identification of positive animals has had in such metrics has been also assessed. Results will help to clarify the dynamics for disease transmission in ACM and to estimate the impact of alternative control strategies in one of the regions of Europe in which the disease is yet to be eradicated.

## Results

### Study population

Through the twelve-year period (1997–2009) under study, 18,056 herd tests and 990,643 individual tests were performed in 1,734 farms of ACM. Most of the tested herds were beef herds (n = 1392, 80.3%), whereas the remaining herds were categorized as dairy (n = 199, 11.5%) and bullfighting (n = 143 herds, 8.2%). Median number of tested animals per round was 29, although it varied with the herd type (Table 1).

**Table 1:** Data (median and interquartile range or proportion) of the 18,056 herd-tests performed on 1,734 herds included in the tuberculosis eradication campaigns in the Autonomous Community of Madrid in 1997–2009

	**Beef**	**Dairy**	**Bullfighting**	**Total**
**Study population**				
Number of herds tested per year	971 (844–1030)	156 (142–162)	75 (60–86)	1193 (1068–1262)
Number of tests/year	1065 (975–1142)	182 (178–233)	85 (67–109)	1333 (1232–1435)
Number of animals tested/round	25 (13–49)	53 (23–105)	74 (33–141)	29 (14–60)
Days between tests (non-bTB)	355 (217–372)	337 (206–376)	328 (203–383)	351 (215–372)
**bTB data**				
% positive herds	22.2	50.3	25.2	25.6
% recurrently infected herds	6.8	34	13.9	13.5
% of outbreaks in OTF farms	29.7	47.2	31.7	38.8
Number of animals tested/round	40 (19–76)	69 (36–131)	89 (53–141)	25 (51–98)
Number of reactors/positive test	2 (1–4)	4.5 (2–11)	3 (1–7.7)	2 (1–6)
Number of outbreaks	330	144	41	515
Number of positive tests/outbreak	1 (1–2)	1 (1–3)	1 (1–2.5)	1 (1–2)
Number of reactors/outbreak	2 (1–5)	5 (2–18)	4 (1–10.5)	3 (1–7)
Number of testing rounds for achievement of OTF status	2 (2–3)	2 (2–4)	2 (2–3)	2 (2–3)
β (test) (median)	2.3 (1.5–4.5)	4.7 (2.2–9.2)	2.2 (1.5–4.5)	2.9 (1.6–6)
β (skin test) (median)	1.94 (1.3–3.1)	4.2 (2–8.4)	2.1 (1.2–3.2)	2.4 (1.5–4.9)
β (skin test + IFN-γ assay) (median)	5.7 (4.4–7.2)	6.4 (4.5–10.5)	4.9 (2.1–6.7)	5.8 (4.4–7.5)
β (herd) (median)*	2.3 (1.5–3.6)	4.3 (2.4–6.3)	2 (1.5–3.5)	2.6 (1.6–4.3)

*After removal of outliers.

### bTB data

At the beginning of the study-period overall herd prevalence in ACM was 27.6%, while in 2009 herd prevalence was 5.54%. There were 515 bTB outbreaks reported in 444 herds, of which 330 (64.07%), 144 (27.96%) and 41 (7.96%) outbreaks were reported in 308 (59.81%), 100 (22.52%) and 36 (8.11%) beef, dairy and bullfighting herds, respectively (Table 1). Median duration of outbreaks was 456 days and the median number of herd tests to obtain OTF status was 2. It is noteworthy that the time to obtain OTF status after an outbreak depended on the initial status of the farm; in farms that had just been included in the program and thus had never reached OTF status, median time to obtain the OTF was 568 days, whereas median time to recover OTF status in outbreaks that occurred in OTF herds was 344 days. Median number of reactors detected per positive herd test and outbreak was 2 and 3 animals, respectively. Data depending on herd type are detailed in Table 1.

Proportions of positive herds through the 12-year period, recurrently infected herds, and outbreaks declared in OTF herds were significantly higher (p < 0.05) in dairy cattle (50.3%, 34%, and 47.2%, respectively) compared to beef (22.2%, 6.8%, and 29.7%) and bullfighting (25.2%, 13.9% and 31.7%) cattle (Table 1).

### Within-herd transmission indicators/coefficient

Median number of reactors per positive herd-test and outbreak were higher in dairy herds (4.5 and 5, respectively) than in beef (2 and 2, respectively) and bullfighting (3 and 4, respectively) herds. The value of β_t_ was computed using information from herd-tests in which date of the previous bTB test was available (n = 543, 66.8%). At the test level, the impact of the introduction of IFN-γ assay in positive herds yielded a statistically significant (Mann–Whitney, p < 0.05) increase in the values of β_t_ for every herd type compared to those calculated when only the SIT test was performed, although this increase was less evident in dairy herds (Table 1).

The β_f_ values computed for 17 (13 beef, 3 dairy and 1 bullfighting) of the 298 positive herds in which this parameter could be estimated were identified as outliers and therefore excluded from the analysis. Nevertheless, their inclusion did not affect the results presented here (data not shown). The computed values of β_f_ fitted log-normal distributions for each herd type (Table 2). Significantly (p < 0.001) higher values of β_f_ were observed in dairy herds compared to beef and bullfighting herds, which showed equivalent β_f_ values (p = 0.47).

**Table 2:** Parameters of Lognormal distribution fitting the intra-herd transmission coefficients estimated at the herd level using data from herds of Madrid in 1997–2009 after exclusion of outliers (n = 17)

**Productive type**	**Number of herds**	**Mean**	**Median**	**Standard deviation**
Beef	181	2.77	2.32	1.81
Dairy	74	4.69	3.82	3.37
Bullfighting	26	2.45	2.11	1.37
Total	281	3.24	2.59	2.40

### Survival analysis

Eighteen and 34 out of the 515 outbreaks detected in the period of study were not included in the survival analysis due to anomalous length values (outliers) or because they were resolved by stamping out respectively. From the remaining 463 outbreaks used for fitting the two univariable Cox proportional hazards models, OTF status had been obtained in 438 (95%) of the affected herds and bTB outbreaks were still ongoing in the remaining 25 (5%) herds at the time of the analysis.

**Figure 1:**
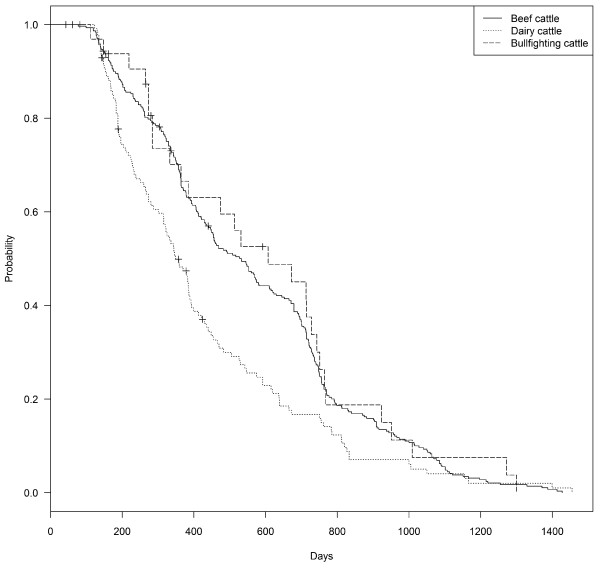
Kaplan-Meier survival estimates of time to recover official tuberculosis free (OTF) status in outbreaks declared on beef (n = 300), dairy (n = 131) and bullfighting (n = 32) herds in the Autonomous Community of Madrid in the period 1997–2009.

Herd type was significantly associated with time to recover OTF status, with outbreaks in dairy herds showing significantly shorter lengths (median 349 days) compared to beef and bullfighting herds (median 532 and 607, respectively), and an increased probability of resolving the outbreak faster, as indicated by the hazard rates (Figure 1, Table 3).

**Table 3:** Results of the univariable survival analyses performed on 463 tuberculosis outbreaks declared in 1997–2009 in the Autonomous Community of Madrid after removal of outliers (n = 18) and outbreaks resolved by herd depopulation (n = 34)

**Survival Analysis**	**Number of outbreaks**	**Resolved outbreaks**	**Median time for recovery of OTF status (days) (95% CI)**	**Probability (hazard ratio) (95% CI)**	***P***
Productive type					
Beef	300	292	532 (453–610)	NA	NA
Dairy	131	118	349 (316–393)	1.46 (1.17-1.81)	<0.001
Bullfighting	32	28	607 (385–751)	0.9 (0.61-1.33)	0.6
Period					
Before 2006	365	365	517 (453–575)	NA	NA
Since 2006	98	73	307 (259–359)	2.18 (1.68-2.83)	<0.001

*NA* non-applicable.

**Figure 2:**
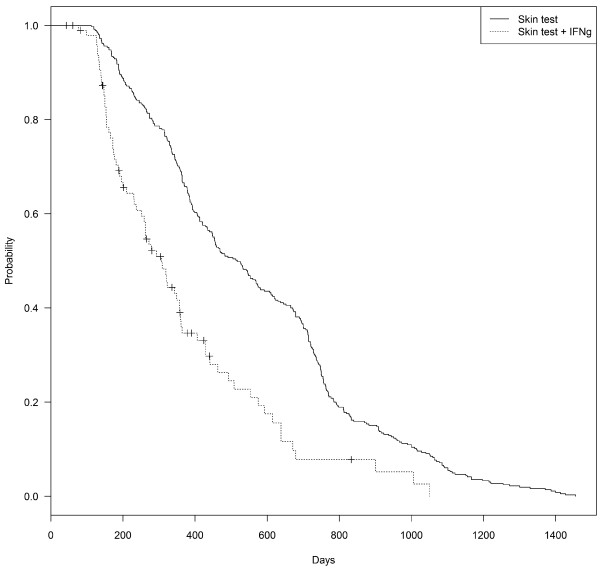
Kaplan-Meier survival estimates of time to recover official tuberculosis free (OTF) status in outbreaks resolved with (n = 98) and without (n = 365) the use of interferon-gamma assay as an ancillary test in the Autonomous Community of Madrid in the period 1997–2009.

Outbreaks managed after implementation of IFN-γ assay in infected herds in 2006 had also significantly shorter duration (median duration 307 days) and increased probability of recovery than those declared before 2006 (median 517 days) (Figure 2, Table 3).

The number of reactors disclosed in the first positive herd test was significantly and inversely associated with the length of the outbreak (hazard ratio 0.98, 95% CI 0.97-0.99) in the multivariable Cox model, whereas hazard ratio for the other two significant variables (herd type and use of IFN-γ assay) did not substantially differ (<15%), compared to the coefficients obtained in the bivariate analyses (data not shown).

Schoenfeld residuals indicated that the assumption of proportional hazards was violated for outbreaks in which time to recover OTF status was shorter than 240 days. Cox models were re-run after elimination of those outbreaks; subsequently, the proportional hazards assumption was met (P = 0.3) and results of the hazards ratios remain unchanged as indicated by the overlapping 95% CI.

## Discussion

Eradication of bTB at a herd level, which is prerequisite for disease eradication at a region and country level, has been impaired by the chronic nature of the disease that can lead to relatively long periods of “silent infection” in infected animals, especially at low infectious doses [8]. This feature of the disease, together with limitations in accuracy of the diagnostic techniques, can substantially extend the time needed for declaring a herd bTB free. Several studies have been published dealing with the identification of risk factors at an individual or herd level associated with bTB [6,7,13-15] or with the detection of predictors of future herd breakdowns in bTB free herds [16], but longitudinal studies aiming at elucidating the disease dynamics in infected herds under a strong eradication pressure such as the one presented here have not been published in the peer reviewed literature. Here, we analyzed data from all cattle herds in ACM included in the Spanish bTB eradication program, therefore providing information on its situation without sampling-biases. ACM was selected for this study due to its high herd-prevalence, its relative small size (which allows for including the entire herd population in the analysis) and the presence of most epidemiological situations described in the rest of the country; these include different herd types, presence of wildlife reservoirs, and extensive and intensively managed herds. Thus, results here are likely to be representative of other Spanish regions. Our data suggest that herd type influences the probability of success of the bTB control program in ACM, and perhaps, in other bTB-infected regions of Spain and Europe.

The median value of β_t_, estimated here was similar to the values reported in Argentina [10], New Zealand [9] and the Netherlands [17], suggesting that, on average, every infectious animal led to 2–5 infections per year. However, the value of β_t_ was significantly higher for dairies compared with other herd types. Dairy farms were at higher risk for bTB than beef and bullfighting herds, as indicated by the larger values of β_t_, proportion of outbreaks, and proportion of recurrently infected farms observed in dairy herds compared to other herd types. This finding may be related to a combination of factors that favor disease spread in these herds including, for example, high contact rates, high density, and presence of stressors associated with the intensive management of animals, as previously suggested [6,7]. Arguably, some animals may have been culled before becoming positive, which would result in an underestimation of beta (especially in beef herds with a higher replacement rate); however, animals culled prior to detection were, consequently, not considered as sources of infection at the subsequent estimation of beta, which would result in an overestimation of the parameter. In addition, animals from bTB-infected cannot be moved to other herds, therefore decreasing the likeness of removal of infected animals from the herd before detection. Imperfect sensitivity was also incorporated to estimate the number of true infected animals. Therefore, estimates here would be, on average, accurate. The number of herd tests required to obtain OTF status was not influenced by the herd type; however, the time to achieve OTF status was shorter for dairy herds compared to beef and bullfighting herds, probably as a consequence of a short duration of the between-test period in dairy farms. In addition, the occurrence of occasional individual infections due to contact with infected wildlife could also contribute to extend duration of outbreaks in extensively managed herds (typically beef and bullfighting herds). These results suggest that a possible herd-type effect should be taken into account when measures for bTB eradication are being implemented.

Introduction of the IFN-γ assay for the identification of infected animals in positive herds resulted in an increase on the values of β_t_ (Table 1), likely as a consequence of an increase in the sensitivity of the detection, as expected when this technique is used as an ancillary test [18,19]. Consequently, use of the IFN-γ assay also decreased significantly the median time to recover OTF status after an outbreak (Table 3). Misclassification of still-infected farms as OTF due to the change in the regulations implemented in 2006 does not seem likely, as only 3 (6%) of the 50 farms that achieved OTF status after 2006 were reinfected by 2010. These findings suggest that incorporation of the IFN-γ was beneficial for the control program in ACM, accelerating bTB eradication at a herd-level.

Interestingly, results of the multivariable model suggest that the larger the number of bTB-positive animals detected at the first herd test, the shorter the time to eradicate the disease from the farm. A large number of positive animals at the first herd test may be interpreted as an indication of low probability of false negative results, thus, decreasing the time of eradication. This finding is consistent with the results obtained by the use of IFN-γ, which most likely increases the sensitivity of the detection.

Outliers were removed from the dataset before the survival analysis (10% outbreaks) and before identifying the theoretical distribution that best fitted the within-herd transmission data (6% herds) because of the uncertainty related to the accuracy of such estimations. There are a number of factors that could lead to unusual lengths of outbreaks and high values of β_f_ (referred to as outliers) including, for example, recording bias, introduction of infected animals, or participation of wildlife reservoirs in the maintenance and transmission of the disease (whose effect on bTB transmission would be included in the same β_f_ estimate). Still, from the decision making perspective, identification and further investigation of such outliers is of interest, because they may represent farms with particular epidemiological conditions that truly increase the risk for the disease, or, alternatively, misreporting of data related with disease identification. Nevertheless, if outliers were not excluded from the analyses the results obtained did not change significantly as shown by the overlapping 95% CI in all estimates (data not shown).

Arguably, the formulation used here to compute the transmission rate is relatively simple, because certain factors that may affect transmission, such as absence of random contact among susceptible and infected animals, management practices, or the potential role that certain wildlife species may play in disease transmission, were not considered in the formulation. Increasing model complexity would require quantitative knowledge on the value that certain epidemiological factors took in the study population that was not available to the authors including, for example, number and distribution of wildlife species or rate of contact between cattle per herd. Most important, it is uncertain whether inclusion of such information in the model formulation would result in significantly different estimates for the transmission rate compared to those presented here. In the absence of such information, estimates presented here may be useful approximations of the true value values of the transmission rate of bovine tuberculosis in Spain that allow to compare the estimates between different herd types and other epidemiological factors. This could aid in the evaluation of the impact of control strategies for different production systems. In any case, the methodological approach presented here may help the administration of control programs to identify and further investigate those farms in which unusually high transmission rates suggest the occurrence of an anomaly in the pattern of disease transmission.

## Conclusions

Results presented here show that herd type influenced the likelihood of success of a bTB control program in an infected region of Spain and that use of the IFN-γ contributed to the success of the control program as a complementary tool in infected herds. Estimates and analytical approaches presented here will help in the design, implementation and evaluation of bTB control programs in ACM and other infected regions of Spain.

## Methods

### Study population

Data from every farm located in ACM and included in the Spanish bTB Eradication Program between 1997 and 2009 (n = 1,734) were analyzed. Recorded information included herd type (beef, dairy, bullfighting), herd size, number of animals tested, and number of positive animals at every round of testing. Management and handling of animals was performed according to Spanish Legislation. No data from experimental research has been included in the study.

TB diagnosis was performed according to specifications provided in the Spanish bTB Eradication Program. Briefly, single intradermal tuberculin (SIT) test was used as the screening technique following European and Spanish legislation (EU Council Directive 64/432/CEE and RD 2611/1996). The SIT test was performed by bovine field practitioners using 0.1 mL (0.1 mg, 25000 CTU) of the official bovine PPD (CZ Veterinaria, Porriño, Spain), whose potency was periodically assessed according to the OIE standards by the Spanish National Reference Laboratory using in-vivo assays, and every >6 weeks old cattle in the herd was tested. For the purpose of the analysis a bTB outbreak was declared in a farm if one or more reactors were disclosed in a herd-test, and ended when the herd obtained the official bTB-free (OTF) status. A recurrently infected herd was defined as a herd in which more than one bTB episode was declared. Although several adjustments were introduced in the eradication program in the 12 year period assessed here (1997–2009), two main stages can be differentiated based on the testing strategy used.

· Before 2006, to obtain OTF status a herd had to undergo two consecutive herd-tests at least 6 months apart in which no reactors were disclosed, and subsequently, be subjected to yearly testing of all > 6 weeks of age animals to maintain the status. To re-gain OTF status in bTB positive herds, the interval between the two consecutive tests was at least 60 days. If reactors were detected in a OTF herd but post-mortem analysis could not confirm the presence of the disease and infection was not suspected based on epidemiological grounds a herd could also recover OTF status after a single negative round-test performed at least 60 days after the removal of the reactors.

· In 2006 the interferon-gamma (IFN-γ) detection assay was introduced as an ancillary test for detection of the maximum number of infected animals in positive herds (in which tuberculosis infection had been confirmed by bacteriology or epidemiological data) to increase the sensitivity of the detection system (as contemplated in the national eradication program for regions of high prevalence as Spain); the test was performed using the same bovine PPD used for skin testing and 0.05 as the cut-off value as described previously [18], and only > 6 months old cattle were tested using the IFN-γ. Herds in which SIT and IFN-γ were applied in parallel could re-gain OTF status if no reactors were detected in two consecutive SIT herd-tests and infection could not be confirmed in any positive animals detected by the IFN-γ assay, and infection was not suspected based on epidemiological grounds. In addition, since 2006 OTF herds had to undergo two annual herd-tests with negative results to maintain their status.

### Data analysis

#### Transmission coefficient

The transmission coefficient, β, was defined as the average number of animals infected from an infectious individual per unit of time [20]. For the purpose of the analysis all transmission was assumed to be bovine-to-bovine. The value of β was computed as the exponential of ln(*C*_*t*_) – ln (*S*_*t*_*I*_*t*_*/N*), as described elsewhere for bTB [10], where I_t_, the number of bTB-infectious animals, was estimated as a function of C_t_ and C_t+1_. C_t_ and C_t+1_ are the number of bTB cases detected in time and t + 1, respectively, and S_t_ is the number of susceptible (non infected) cattle in time t, and time t and t + 1 indicate, respectively, two consecutive herd tests. The values of C_t_ and C_t+1_ were computed considering the number of bTB-positive results and assuming that the sensitivity and the specificity of the diagnostic test ranged from 63.2 to 100% and 75.5 to 99% respectively, with 83.9% and 96.8% as most probable values for each of them [21]. Thus, a Pert distribution was used to model the uncertainty about the true value of the test sensitivity and specificity and the values of C_t_ and C_t+1_ were computed following a stochastic process. Because it is uncertain how many of the bTB-infected animals were infectious and because bTB-positive reactors were immediately removed from the herd, I_t_ was assumed to follow a uniform distribution with parameters 1 and C_t_, which represent the extreme scenarios in which only one and every infected animal were infectious, respectively. If over the course of an outbreak there was a herd test with no positive reaction, followed by a herd test with positive results, a value of I_t_ = 1 was used, i.e., assuming that there was one infected animal in the farm that was not detected by the test. Values of β were computed after 1000 simulations using @RISK 5.5 (Palisade Co, Ithaca, NY, USA). Median β values were estimated for each period between consecutive herd tests (β_t_) and for each farm (β_f_) when time since the last herd test performed was available. Outliers in the distribution of β_t_ and β_f_ per herd type were detected as described below and removed because authors suspect that were associated with information bias. The theoretical distribution that best fitted (P > 0.05) the observed distribution of β_f_ was identified for each herd type using a Chi-square test implemented in the @RISK software. Association between β_f_ and herd type was explored using a Kruskal-Wallis test, which was implemented in the SPSS software (IBM Inc, Chicago, Il, USA).

#### Probability of bTB re-infection and time to recover official bTB-free status

The association between the probability of suffering more than one bTB outbreak and herd type was quantified using a Chi-square test.

Life tables were created to display the probability of OTF recovery by productive type in infected herds. Start of the at-risk period was set by the first positive herd-test in a farm, and test date at which the farm recovered OTF status was used as the failure time. Censoring time was fixed at the last herd-test recorded for those farms in which outbreaks could not be solved.

The association between herd type and time for recovery of OTF status after detection of an outbreak was explored using a Cox proportional hazards model after removal of outbreaks showing anomalous length values, which were identified as outliers if lying more than 1.5*interquartile range (IQR) from the first or third quartile. Another Cox proportional hazards model was fitted to compare official bTB-free status recovery times before and after the implementation of the IFN-γ in 2006. Additionally, a multivariable Cox proportional hazards model was also fitted using herd type, use of IFN-γ assay (yes, no), number of reactors disclosed in the initial test, and β coefficient as predictor variables. The assumption of proportional hazards in the Cox tests was assessed by computing Schoenfeld residuals [22].

Proportional hazards models were implemented using the R software [23,24].

## Authors' contributions

JA performed the statistical analyses and drafted the manuscript, AP participated in the data analyses and manuscript preparation and editing, JB and CC gathered the data from the official database systems and performed descriptive analyses, BR and SR carried out the IFN-γ tests, JLS, RD and JC coordinated the eradication campaigns, designed the databases and revised critically the manuscript, LdJ and LD designed the study and coordinated the units involved in it. All authors read and approved the final manuscript.
